# The Influence of Macrophytes on Sediment Resuspension and the Effect of Associated Nutrients in a Shallow and Large Lake (Lake Taihu, China)

**DOI:** 10.1371/journal.pone.0127915

**Published:** 2015-06-01

**Authors:** Mengyuan Zhu, Guangwei Zhu, Leena Nurminen, Tingfeng Wu, Jianming Deng, Yunlin Zhang, Boqiang Qin, Anne-Mari Ventelä

**Affiliations:** 1 State Key Laboratory of Lake Science and Environment, Nanjing Institute of Geography and Limnology, Chinese Academy of Sciences, Nanjing, 210008, China; 2 Department of Biological and Environmental Sciences, University of Helsinki, Helsinki, 00014, Finland; 3 Pyhäjärvi Institute, Kauttua, 27500, Finland; Northwest Fisheries Science Center, NOAA Fisheries, UNITED STATES

## Abstract

A yearlong campaign to examine sediment resuspension was conducted in large, shallow and eutrophic Lake Taihu, China, to investigate the influence of vegetation on sediment resuspension and its nutrient effects. The study was conducted at 6 sites located in both phytoplankton-dominated zone and macrophyte-dominated zone of the lake, lasting for a total of 13 months, with collections made at two-week intervals. Sediment resuspension in Taihu, with a two-week high average rate of 1771 g·m^-2^·d^-1^ and a yearly average rate of 377 g·m^-2^·d^-1^, is much stronger than in many other lakes worldwide, as Taihu is quite shallow and contains a long fetch. The occurrence of macrophytes, however, provided quite strong abatement of sediment resuspension, which may reduce the sediment resuspension rate up to 29-fold. The contribution of nitrogen and phosphorus to the water column from sediment resuspension was estimated as 0.34 mg·L^-1^ and 0.051 mg·L^-1^ in the phytoplankton-dominated zone. Sediment resuspension also largely reduced transparency and then stimulated phytoplankton growth. Therefore, sediment resuspension may be one of the most important factors delaying the recovery of eutrophic Lake Taihu, and the influence of sediment resuspension on water quality must also be taken into account by the lake managers when they determine the restoration target.

## Introduction

In shallow lakes, sediment resuspension is a very common phenomenon and has an important influence on the ecosystem. Particles resuspended from the bottom sediment may increase turbidity and deteriorate the underwater light field [1–3]. The resuspension process influences nutrient flux at the sediment-water interface and in the water column [4–6], and then affects primary production by macrophytes and phytoplankton [7,8]. Suspending nutrients from the sediment increase phytoplankton biomass, delaying the recovery of eutrophic lake ecosystems [9,10], and thus considerable attention is paid to sediment resuspension in the management of water quality.

Previous studies have shown that the intensity of sediment resuspension is influenced by many factors, including lake shape, water depth, sediment quality, wind-induced wave action and macrophyte coverage [11,12]. Shallow and large lakes are especially prone to sediment resuspension due to wind-induced wave action [12–15], and seasonal variation in wind-wave processes may substantially influence the yearly sediment resuspension rate. Wind-induced wave was considered the more important factor influencing sediment resuspension than currents, especially under strong wind forcing, which could easily result in sediment resuspension [15–18]. The existence of aquatic macrophytes is also a key factor influencing sediment resuspension in shallow lakes [19], because of the effect of vegetation on hydrodynamics as macrophytes substantially moderate the effect of wind waves [20].

Due to the variety of factors affecting resuspension, there is substantial between-lake as well as spatial and temporal within-lake variation in sediment resuspension [11,21,22]. The dynamic ratio (the square root of lake surface area in square kilometers divided by the average depth in meters) has been used in analyzing the relationship between wave disturbance and sediment resuspension [9]. According to studies in 36 Florida lakes [23], lakes with dynamic ratios above 0.8 are prone to sediment resuspension.

Lake Taihu (referred as “Taihu” below for short) is very shallow, with a maximum depth of less than 3 meters and an average depth of 1.9 meters, and has a large surface area of 2338 km^2^ [24]. Taihu thus has a relatively high dynamic ratio (25.4) compared with many other lakes [25]. Earlier studies in Taihu indicated that sediment resuspension in this large and shallow lake played a significant part in chemical and biological processes [26].

Eutrophication has been a big problem in Taihu for several decades [24] to the point where harmful algal blooms caused a drinking water crisis in 2007, which affected two million people [27,28]. This serious event has raised considerable concern resulting in efforts to control phytoplankton blooms, but the complicated environmental processes driving these blooms, including sediment resuspension, have made the effort difficult [28,29].

Taihu has two distinctly different ecotypes, which are phytoplankton- and macrophyte-dominated areas. These two ecotypes in Taihu have significantly different turbidity levels and underwater light fields [30], which indicate differences also in sediment resuspension. However, previous studies on sediment resuspension in Taihu have been conducted mostly in the phytoplankton-dominated zone, but seldom in the macrophyte-dominated zone, or only with short-term observations [26,31,32], and thus may not represent a comprehensive situation of sediment resuspension processes, nor predict the ecological effects of these processes.

As sediment resuspension is an important factor regulating internal nutrient loading, the development and coverage of macrophytes may be important in the overall nutrient flux of shallow water bodies [33]. Quantitative methods to evaluate resuspension processes in Taihu are still lacking and no comprehensive studies on the influence of macrophytes on sediment resuspension have been conducted which cover all seasons. As noted above earlier observations concerning sediment resuspension mechanisms and subsequent control methods in other lakes may not be suitable for Taihu [34–36], because of different dynamic ratios or driving factors for resuspension in different lakes, or even in different parts of the lake [37].

Therefore, to understand the linkage between the sediment-water interaction and resuspension-originated nutrient loading that causes massive phytoplankton blooms and deteriorate water quality, a comprehensive spatial and temporal study on continuous sediment resuspension processes is of interest. In this study, 6 sites were chosen that were spatially distributed in the two different ecotypes, phytoplankton- and macrophyte-dominated, and with different distances from the lakeshore in Taihu. The study duration was more than one year so as to cover all seasons, including a typhoon period with high winds. This study aimed to quantify the influence of vegetation on sediment resuspension and to quantity resuspension-borne phosphorus and nitrogen loading.

## Material and Methods

### Ethics statement

No specific permits were required for the described field studies. The location studied is not privately-owned or protected in any way, and the field studies did not involve endangered or protected species.

### Observation sites

**Fig 1:**
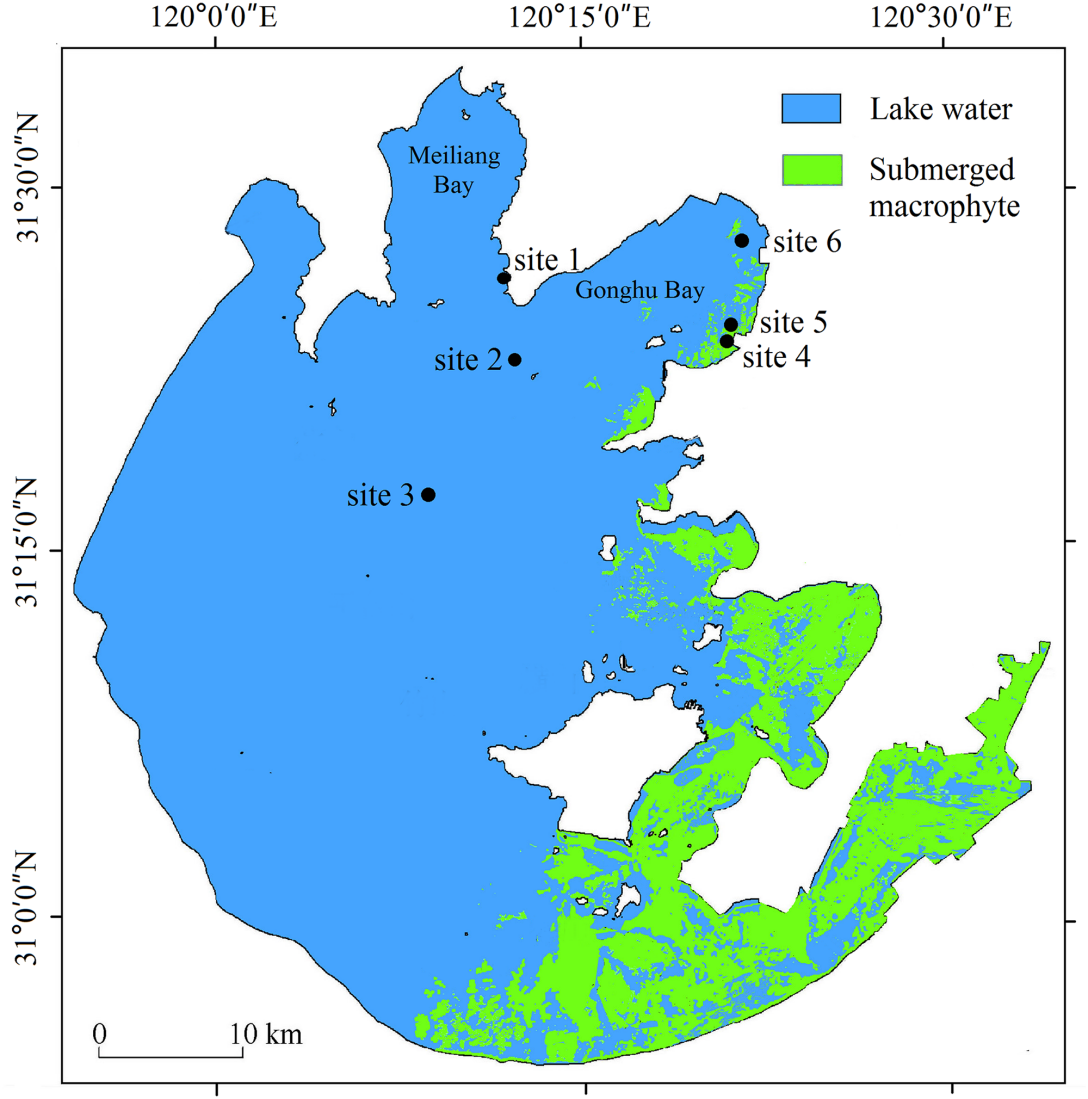
Map of Taihu and 6 observation sites for the trap experiment. Distribution of submerged macrophytes was provided by Dong [38].

Sediment resuspension measurements were conducted at 6 sites ranging from lakeshore to open water in both the phytoplankton-dominated and macrophyte-dominated zones (Fig 1). Site 1 was located near the field station of Taihu Laboratory for Lake Ecosystem Research (TLLER) in Meiliang Bay, 250 m from the lakeshore. Site 3 was located in the central part of the lake, while site 2 was located between sites 1 and 3. Sites 1, 2 and 3 represent a transect from the lake shore to open water in the phytoplankton-dominated zone. Meiliang Bay is one of the most polluted regions in Taihu, with the earliest phytoplankton bloom occurring here in 1980s [39].

Sites 4, 5 and 6 were all located in Gonghu Bay, representing areas with full macrophyte coverage, semi-macrophyte coverage and no macrophyte coverage, respectively. Site 4 was located 800 m from the eastern lakeshore and nearly 1500 m from the southern lakeshore, with 100% macrophyte coverage (*Potamogeton maackianus*, *Potamogeton malaianus*, *Nymphoides peltatum*, *Trapa incisa*, *Ceratophyllum demersum*, *Myriophyllum spicatum* and *Hydrilla verticillata*). Site 5 was located 1000 m from the eastern lakeshore and 1400 m from the southern lakeshore, with 60% (visual estimation) macrophyte coverage, with similar species composition as site 4. At sites 4 and 5, macrophytes sprout in late March, grow to the water surface in May, and wither from October until December. Site 6 was located in the central part of the bay bottom, where phytoplankton blooms have extended in recent decades causing a gradual disappearance of macrophytes [40]. Therefore, site 6 in this study was sorted with the sites from phytoplankton-dominated zone, to show the differences between macrophyte covered area (sites 4 and 5) and no macrophyte covered area (site 6) at the same bay (Table 1).

**Table 1:** Ecotype, water depth and lake water quality at each site during the high productive and the low productive seasons.

Sites		1	2	3	4	5	6
Ecotype		PD ^a^	PD	PD	MD ^b^	MD	PD
**WD** ^c^ (m)	HG ^d^	1.6±0.2	2.0±0.5	2.4±0.3	1.4±0.3	1.4±0.3	1.5±0.3
	LG ^e^	1.4±0.2	1.6±1.0	2.3±0.6	1.3±0.3	1.3±0.2	1.5±0.3
**Chl**-*a* ^f^ (μg∙L^-1^)	HG	34±34	32±31	21±22	5±4	6±8	15±11
	LG	8±3	9±2	8±3	5±2	6±2	9±5
**SS** ^g^ (mg∙L^-1^)	HG	59±45	67±31	69±28	3±2	8±6	46±21
	LG	36±23	78±66	111±105	14±12	17±11	35±16
**LOI** ^h^ (%)	HG	30±12	30±17	27±15	85±15	50±19	25±10
	LG	29±12	20±8	16±5	44±17	32±10	27±8
**TP** ^i^ (mg∙L^-1^)	HG	0.132±0.075	0.126±0.080	0.112±0.060	0.025±0.012	0.028±0.011	0.103±0.034
	LG	0.064±0.013	0.092±0.019	0.116±0.032	0.036±0.019	0.035±0.014	0.098±0.029
**TN** ^j^ (mg∙L^-1^)	HG	1.83±0.77	2.03±0.85	1.99±0.73	0.66±0.16	0.80±0.32	1.87±0.83
	LG	2.77±0.69	3.52±1.09	3.36±0.69	2.04±0.94	1.96±0.66	2.85±0.46

^a^ “PD” indicates phytoplankton dominated;

^b^ “MD” indicates macrophyte dominated;

^c^ “WD” indicates water depth;

^d^ “HG” indicates high productive season;

^e^ “LG” indicates low productive season;

^f^ “Chl-*a*” indicates chlorophyll *a* concentration;

^g^ “SS” indicates suspended solids concentration;

^h^ “LOI” indicates loss on ignition;

^i^ “TP” indicates total phosphorus concentration;

^j^ “TN” indicates total nitrogen concentration.

### Sediment resuspension observation

Sediment resuspension was measured using triplicate plexi-glass traps (30 cm long and 5 cm internal diameter) at each site. The upper “mouth” of the traps was fixed at 0.5 m above the sediment, with weights at the bottom to keep them vertical.

Mixtures of water and sediment in traps were collected every 14 to 16 days (depending on weather) from 5 August 2012 to 2 September 2013, resulting in a total of 27 sampling points. All the contents within the traps, including sediment, water, and organic detritus, were collected and taken back to the laboratory to determine suspended solids (SS), total nitrogen (TN) and total phosphorus (TP) concentrations (see details below). Additionally, sediment from the traps was also collected to measure particle size distribution.

On the first sampling date (5 Aug 2012) surface sediment was collected by a grab sampler to determine TN, TP, total organic carbon (TOC) content, water content and particle size distribution.

Simultaneously with trap collection, water samples at the surface of the water column were collected to determine SS, TN, TP, dissolved total nitrogen (DTN), dissolved total phosphorus (DTP) and planktonic chlorophyll *a* (Chl-*a*) concentrations. Water temperature was measured *in situ* at the surface of the water column with a Yellow Springs Instruments (YSI) 6600 V2 multi-sensor sonde (YSI Inc., USA).

High-frequency wind speed and direction data (10 min interval) at site 1 were acquired from sensors associated with Global Lake Ecosystem Observation Network (GLEON). Unfortunately, data from 1 Apr 2013 to 12 May 2013 were lost. High-frequency wind speed and direction data (15 min interval) near sites 4 and 5 were recorded from November 2012 onwards by a Hobo automatic recording weather station (Onset Computer Corporation, USA). Wind speed and direction at sites 2, 3 and 6 was not recorded.

### Laboratory analysis

SS concentrations and organic carbon content in water samples were measured by weighing the GF/F glass membrane after collection of a known volume (v_w_) of water samples. New membranes were pre-combusted at 550°C for 4 hours then weighed (recorded as weight_1_, w_1_). After filtration the membrane was first dried at 105°C for 4 hours then weighed and recorded as weight_2_ (w_2_). The filter was subsequently combusted at 550°C for 4 hours, weighted and recorded as weight_3_ (w_3_). Suspended solids were determined from SS = (w_2_ - w_1_) / v_w_, while the organic carbon content was determined depending on loss on ignition (LOI) [41], and LOI = (w_2_ - w_3_) / (w_2_ - w_1_).

DTP concentration in filtered water (through GF/F glass membrane) and TP concentration in unfiltered water were determined by spectrophotometry at a wavelength of 700 nm following the molybdenum blue method, after digestion with alkaline potassium persulfate (K_2_S_2_O_8_ + NaOH) [42]. DTN concentration in filtered water and TN concentration in unfiltered water were measured by spectrophotometry at 210 nm after digestion [42].

Approximately 10 g (wet weight) sediment was air-dried under room temperature for one week, and weighed before and after air-drying to calculate the water content. The particle size of sediment was determined with laser particle size analyzer (Malvern Instrument, UK) after acidized in dilute hydrochloric acid and hydrogen peroxide. Air-dried sediments were ground into powder until they could pass a 150-μm mesh. Approximately 20 mg powder was weighed and put into 25 mL deionized water to determine TN and TP concentrations with the same method as used for the water samples [43]. The powder was also used to determine TOC content using the potassium dichromate method according to the national standard method in China (GB 7857–87) [44].

Chl-*a* concentrations were determined by spectrophotometry at wavelengths of 665 nm and 750 nm, following extraction with hot 90% ethanol [45].

### Data analysis

Resuspended sediment was estimated by the proposal of Gasith [46], using equations as follows:

R′=S′−T′

$$R\prime \times f_{R}=S\prime \times f_{S}-T\prime \times f_{T}$$

$$R\prime =S\prime \times \frac{f_{S}-f_{T}}{f_{R}-f_{T}}$$

Here, *R’* = resuspended sediment (mg, dry weight); *S’* = entrapped settling flux (mg, dry weight); *T’* = suspended tripton (mg, dry weight); *f*_*R*_ = organic fraction of surface sediment (%); *f*_*S*_ = organic fraction of entrapped material (%); *f*_*T*_ = organic fraction of suspended tription (%).

In this study, *f*_*T*_ was much higher than *f*_*R*_, so the proposal of Gasith was reliably used. Sediment resuspension rate (R) and gross sedimentation rate (S) were calculated using resuspended sediment (g) and entrapped settling flux (g) divided by base area of traps (m^2^) and the observation days (d).

TN and TP resuspension rates were calculated using formula as follows:

$$TN_{R}=\left(TN_{T}-DTN_{lw}\right)\times V_{T}\times \frac{R/S}{A_{t}\times D}$$

$$TP_{R}=\left(TP_{T}-DTP_{lw}\right)\times V_{T}\times \frac{R/S}{A_{t}\times D}$$

Here, *TN*_*R*_ = TN resuspension rate (g·m^-2^·d^-1^); *TN*_*T*_ = TN concentration in traps (mg·L^-1^); *DTN*_*lw*_ = DTN concentration in the water column (mg·L^-1^); *TP*_*R*_ = TP resuspension rate (g·m^-2^·d^-1^); *TP*_*T*_ = TP concentration in traps (mg·L^-1^); *DTP*_*lw*_ = DTP concentration in the water column (mg·L^-1^); *V*_*T*_ = water volume in traps (L); *R/S* = the ratio of sediment resuspension rate to gross sedimentation rate (%); *A*_*t*_ = base area of traps (m^2^); *D* = the observation days (d).

The influence of TN and TP resuspension rates from the sediment on TN and TP concentrations in the water column was estimated in both the phytoplankton- and macrophyte-dominated zones. The increased nutrient concentrations in the water column was calculated using nutrient resuspension rates multiplied by the lake areas in each zone, and then divided by the water volumes in each zone. The averaged area of the macrophyte-dominated zone in Taihu from 2007 to 2010 was approximately 341 km^2^ (nearly 15% of the whole lake area) [47], and the rest of the lake area 1997 km^2^, was considered as the phytoplankton-dominated zone. The averaged water volumes in phytoplankton- and macrophyte-dominated zones were approximately 5.4 and 0.9 billion m^3^ [48].

High frequency wind speed (10 or 15 min interval) was averaged into daily wind speed. According to earlier studies, Luo et al. [49] found the minimum wind speed for sediment resuspension in Taihu to be 5–6 m·s^-1^, and Zhang et al. [50] also indicated that critical wind speed of sediment resuspension in Taihu is between 5 and 6.5 m·s^-1^. Therefore, in this study 6 m·s^-1^ was set as the critical wind speed for sediment resuspension. For every period of wind with current wind speed higher than 6 m·s^-1^ and lasting more than 1 hour, an average wind speed (m·s^-1^) was calculated and multiplied with the duration (s). “Effective wind” for each observed period was calculated as the summation of those products (km), and used to represent strong wind causing sediment resuspension. Some high frequency data at site 1 during Apr and May 2013 was lost so effective wind at site 1 was calculated only for 24 observation periods. While for sites 4 and 5, high frequency wind speed data was recorded from November 2012 onwards, thus effective wind was calculated for 20 periods. There is no effective wind data at sites 2, 3 and 6, and therefore correlations between effective wind and resuspended sediment were only conducted at sites 1, 4 and 5.

Average values and standard errors for Chl-*a*, SS, TN and TP concentrations in the water column, sediment and nutrient resuspension rates were calculated with Microsoft Excel 2010. The correlation analyses (between SS concentration and LOI, between TN or TP and Chl-*a* concentrations, and between effective wind and resuspended sediment) were performed by Pearson Correlation with SPSS (Statistical Program for Social Sciences) 13.0 software. The differences comparison analyses (between indicators in zones with macrophyte coverage and void of macrophyte, and between indicators in different seasons) were analyzed by Wilcoxon signed rank test of non-parametric test with SPSS 13.0 software, and significance levels reported as not significant (p ≥ 0.05), significant (0.01 ≤ p<0.05) or highly significant (p < 0.01).

## Results

### Lake water quality

**Fig 2:**
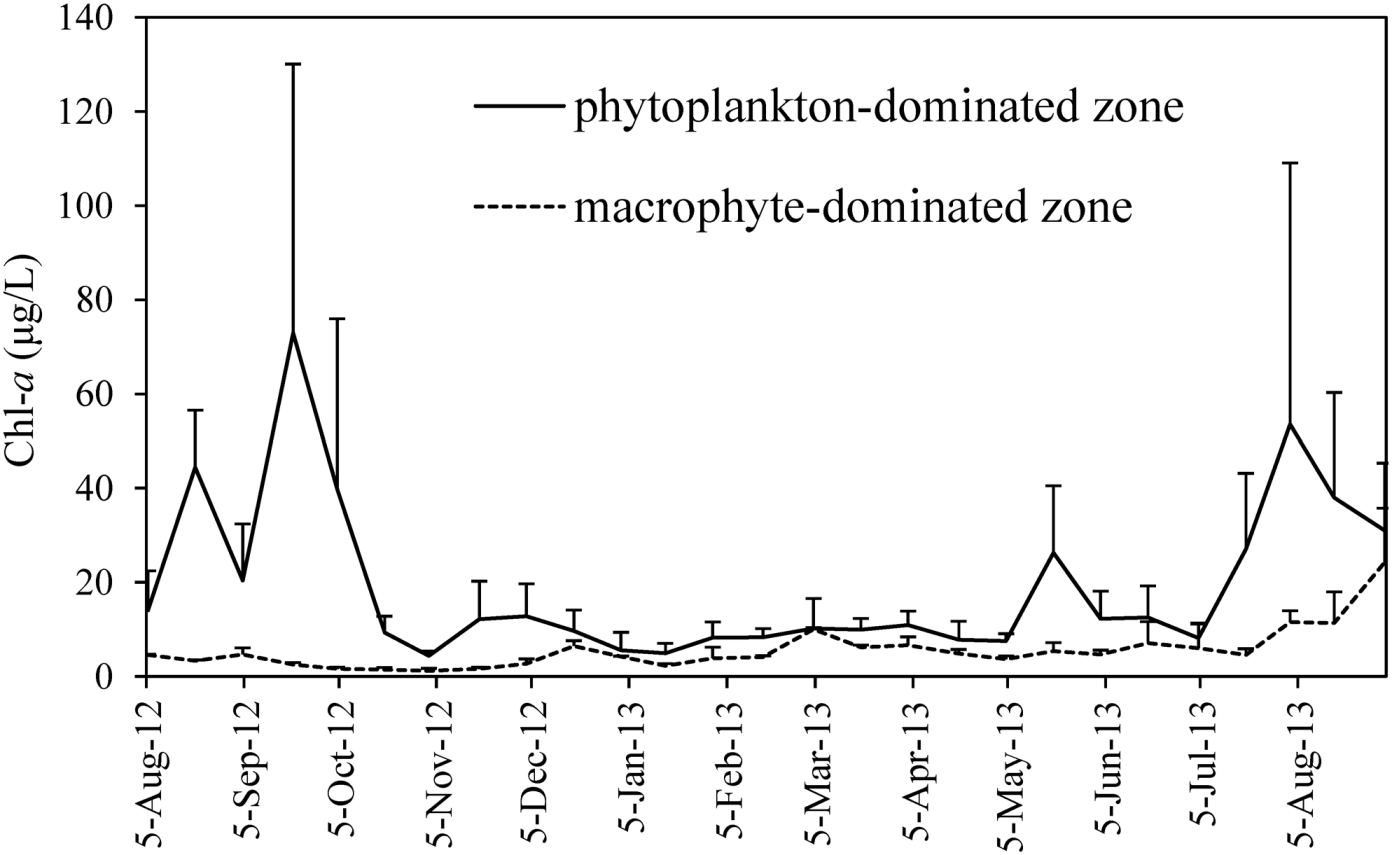
Chlorophyll *a* concentrations at each site in the water column of phytoplankton- and macrophyte-dominated zone during the observation period.

The averaged water depths during the observation year were 1.5, 2.0, and 2.4 m at sites 1, 2, and 3, and 1.4, 1.4, and 1.5 at sites 4, 5, and 6, respectively (Table 1). Chl-*a* concentrations showed highly significant difference (*Z* = −6.078, *p* < 0.01) between the phytoplankton-dominated zone (sites 1, 2, 3, and 6) and macrophyte-dominated zone (sites 4 and 5) (Fig 2). The sampling periods from 5 Aug 2012 to 3 Dec 2012 and from 5 May 2013 to 1 Sep 2013, were determined as “high productive season”, when Chl-*a* concentrations were significantly higher than the other period (*Z* = −4.298, *p* < 0.01, Table 1) in the phytoplankton-dominated zone, with water temperature 24±7°C. The other sampling period from 4 Dec 2012 to 4 May 2013 was determined as the “low productive season”, with water temperature 9±5°C. The percentage of organic matter (LOI) in SS concentration had highly significant negative correlations with SS concentrations (*R* ^*2*^ = 0.272, *p* < 0.01, Table 1). TP concentration correlated significantly with Chl-*a* (*p* < 0.01 at all the 6 sites), while TN concentration did not show correlation with Chl-*a* (*p* > 0.05 at all the 6 sites), having the highest value in April and the lowest in August (Table 1).

### Sediment properties

With full macrophyte coverage, sediment at site 4 showed the highest water content and TOC content, and sediment at site 2 had the lowest water and TOC content (Table 2). Water and TOC content at other 4 sites were similar, around 50% and 1%, respectively. Sediment at site 4 also showed the highest TN content, almost twice as high as at other sites (Table 2). TP content in the sediment at each site varied, with the highest value at site 6 and lowest at site 5 (Table 2).

**Table 2:** Sediment properties at each site during the observation.

Sites	Water content (%)	Total nitrogen (mg·kg^-1^)	Total phosphorus (mg·kg^-1^)	Total organic carbon (%)
**1**	53 ± 1	2526 ± 338	504 ± 75	1.19 ± 0.04
**2**	30 ± 0	1992 ± 168	534 ± 28	0.35 ± 0.08
**3**	54 ± 0	2031 ± 101	372 ± 1	0.98 ± 0.07
**4**	77 ± 0	4496 ± 136	385 ± 7	4.48 ± 0.06
**5**	51 ± 0	1900 ± 123	312 ± 2	1.07 ± 0.09
**6**	47 ± 0	2149 ± 176	660 ± 50	0.97 ± 0.01

### Sediment resuspension rate

**Fig 3:**
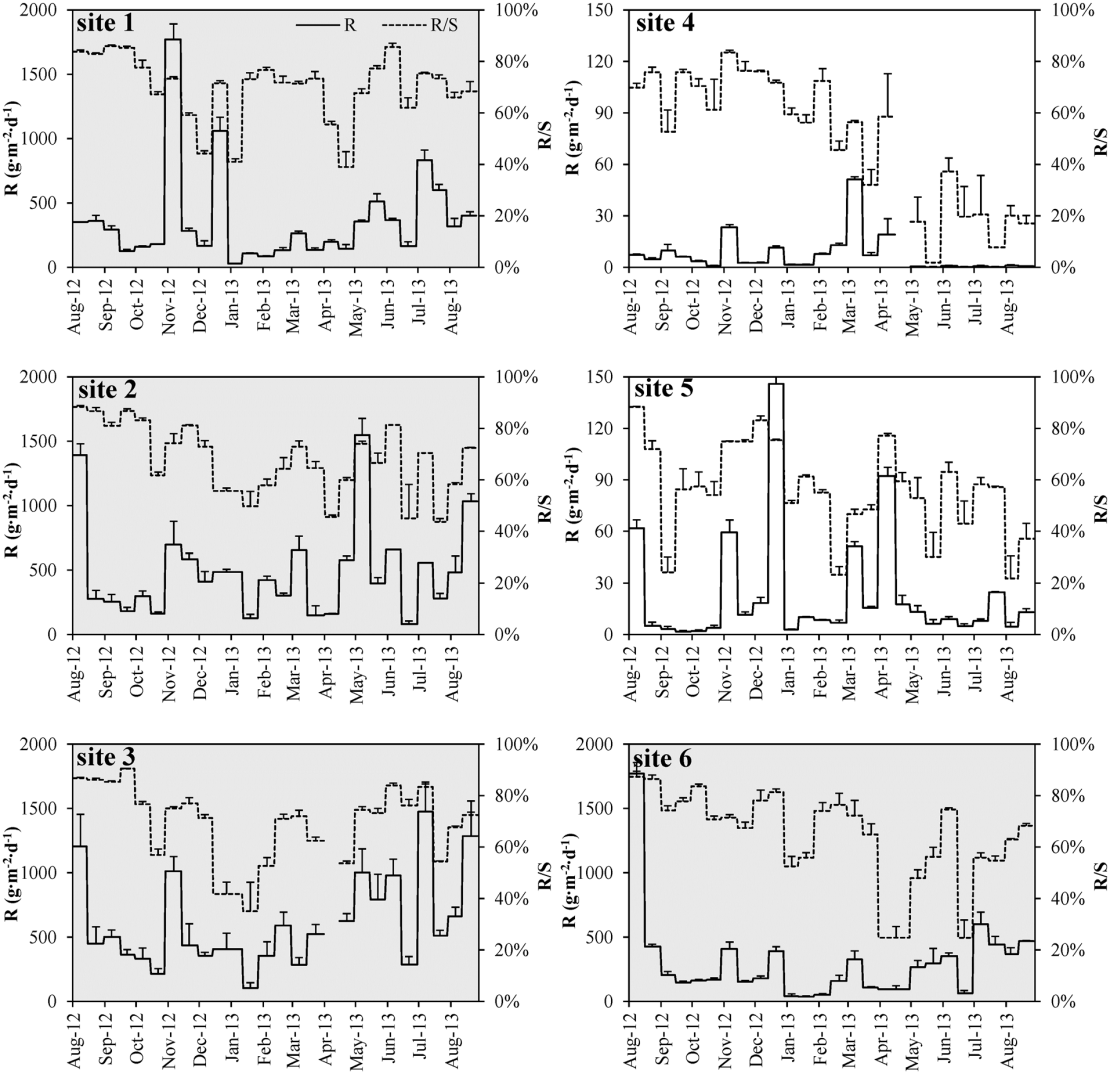
Sediment resuspension rate (R) and the ratio of resuspension to gross sedimentation (R/S) at each site during the observation period. Figures of sites 1, 2, 3 and 6 with gray shading have the same value for y-axis, and figures of sites 4 and 5 have smaller value for y-axis.

The relationship of annual average sediment resuspension rate at each site was: site 3 > site 2 > site 1 > site 6 > site 5 > site 4 (Fig 3). Sediment resuspension rate in the phytoplankton-dominated zone was much higher than in the macrophyte-dominated zone and showed a clear increase from the lakeshore sites to the offshore sites. The annual average sediment resuspension rates at sites 1, 2, 3, and 6 were 363±370 g·m^-2^·d^-1^, 486±355 g·m^-2^·d^-1^, 607±352 g·m^-2^·d^-1^, and 300±330 g·m^-2^·d^-1^, respectively, while only 7±11 g·m^-2^·d^-1^ and 24±33 g·m^-2^·d^-1^ at sites 4 and 5. Sediment resuspension rate at sites void of macrophytes (sites 1, 2, 3 and 6) showed highly significant difference (*Z* = −6.275, *p* < 0.01) with the rates in macrophyte covered area (sites 4 and 5).

Sediment resuspension rate showed highly significant seasonal variation (*Z* = −2.879, *p* < 0.01) between the high productive and low productive season in the phytoplankton-dominated zone. The average sediment resuspension rates at sites 1, 2, 3, and 6 were higher during the high productive season (447±395 g·m^-2^·d^-1^, 555±418 g·m^-2^·d^-1^, 722±383 g·m^-2^·d^-1^, and 396±382 g·m^-2^·d^-1^, respectively) than during the low productive season (231±281 g·m^-2^·d^-1^, 377±178 g·m^-2^·d^-1^, 409±152 g·m^-2^·d^-1^ and 148±115 g·m^-2^·d^-1^, respectively). In the macrophyte-dominated zone, average sediment resuspension rates at sites 4 and 5 during the high productive season (4±6 g·m^-2^·d^-1^, 15±18 g·m^-2^·d^-1^) were much lower than during the low productive season (13±15 g·m^-2^·d^-1^, 38±45 g·m^-2^·d^-1^), but not showing significant variation (*Z* = −1.763, *p* > 0.05).

Similar to the sediment resuspension rates, the R/S ratio was also higher in the phytoplankton-dominated zone (Fig 3). The ratio of organic matter in trapped sediment (O/S) showed a negative relationship with sediment resuspension rate. The annual average O/S ratios were 9%±3% at phytoplankton-dominated zone, and 41%±22% and 19%±10% at sites 4 and 5. The O/S ratio at site 4 which was fully covered with macrophytes was much higher than all the other sites.

The annual average particle size of trapped sediment at each site was similar. The median particle sizes of trapped sediment were 8.4±1.6 μm and 8.0±2.5 μm in the phytoplankton-dominated and macrophyte-dominated zone, respectively.

### Nutrients resuspension rate

**Fig 4:**
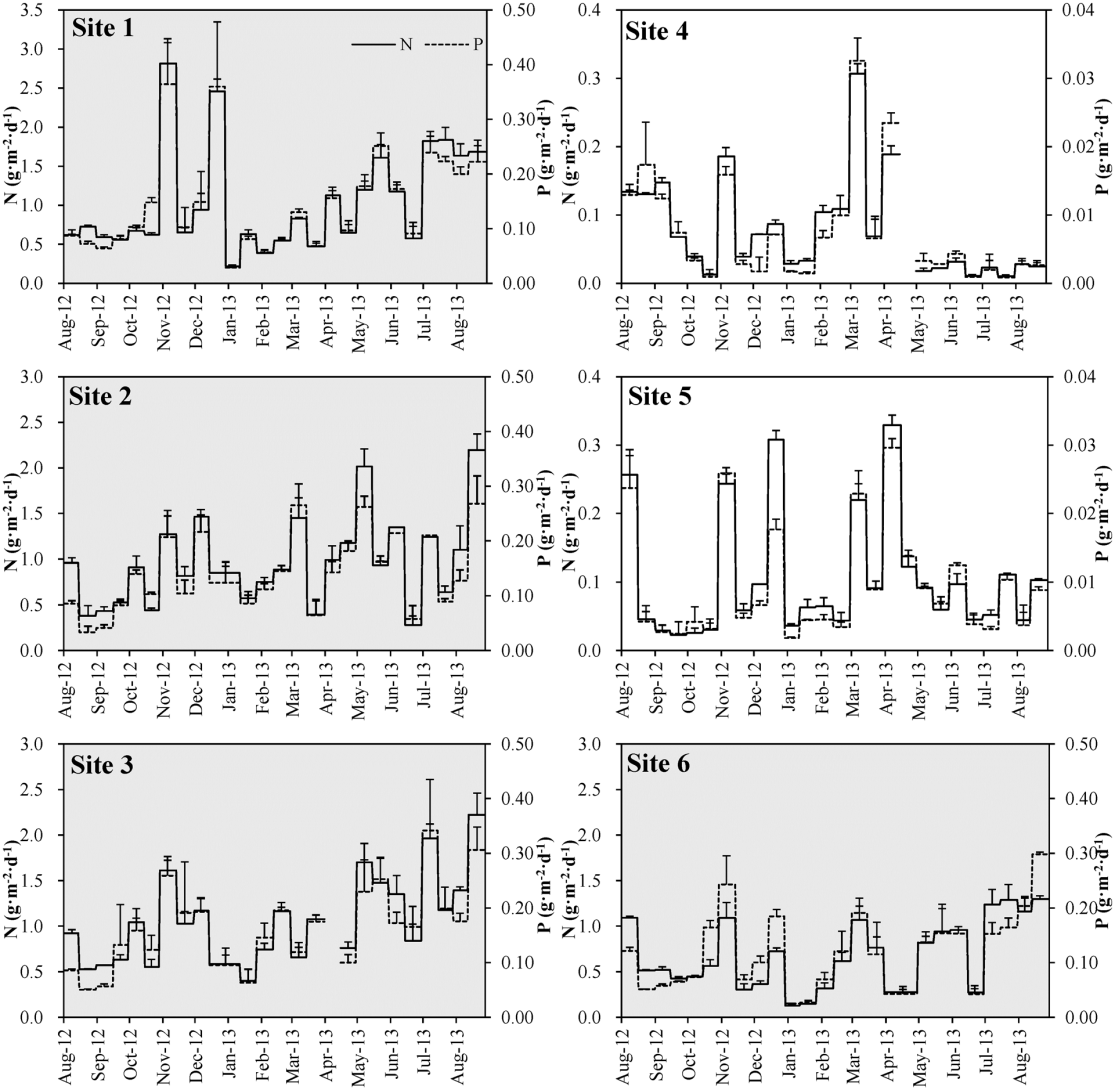
Nitrogen (N) and phosphorus (P) resuspension rate at each site during the observation period. Figures of sites 1, 2, 3 and 6 with gray shading have the same value for y-axis, and figures of sites 4 and 5 have smaller value for y-axis.

**Fig 5:**
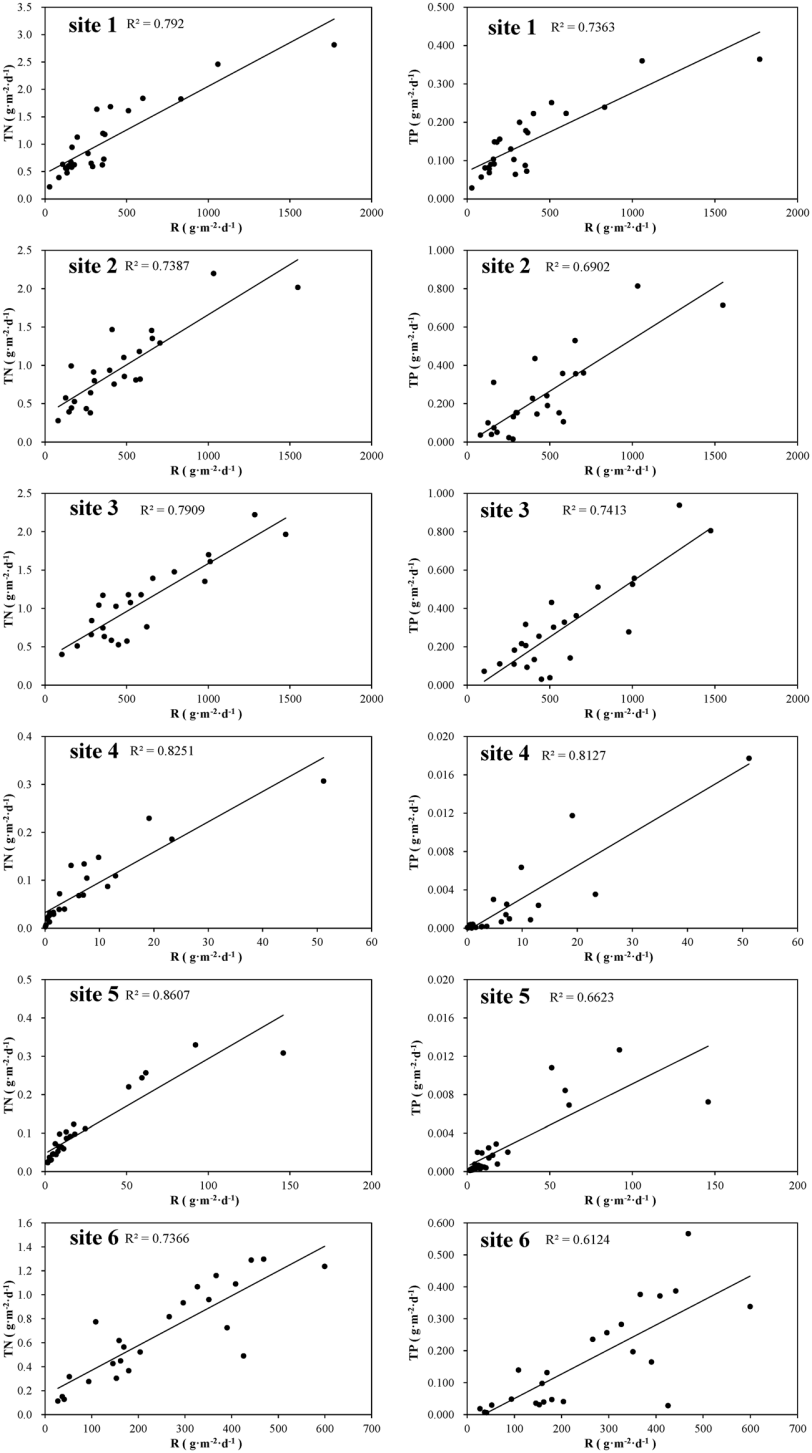
Correlation between sediment resuspension rate and total nitrogen (TN) or total phosphorus (TP) resuspension rate at each site during the observation period.

The relationship of annual average TN and TP resuspension rate at each site was: site 3 > site 1 > site 2 > site 6 > site 5 > site 4 (Fig 4). Both TN and TP resuspension rates showed highly significant correlations with sediment resuspension rates (Fig 5, *p* < 0.01 at all the 6 sites).

The average TN resuspension rates in the phytoplankton-dominated zone were 1.04±0.66 g·m^-2^·d^-1^, 0.96±0.48 g·m^-2^·d^-1^, 1.05±0.47 g·m^-2^·d^-1^, and 0.68±0.37 g·m^-2^·d^-1^ at sites 1, 2, 3 and 6, respectively, while considerably lower 0.08±0.07 g·m^-2^·d^-1^ and 0.11±0.09 g·m^-2^·d^-1^ at sites 4 and 5, increasing significantly from the area of highest macrophyte coverage to the area void of vegetation (*Z* = −5.646, *p* < 0.01).

The average TP resuspension rate showed the same trend with TN resuspension rate (Fig 4). In the phytoplankton-dominated zone, the TP resuspension rates were 0.146±0.087 g·m^-2^·d^-1^, 0.140±0.068 g·m^-2^·d^-1^, 0.164±0.074 g·m^-2^·d^-1^ and 0.118±0.070 g·m^-2^·d^-1^ at sites 1, 2, 3 and 6, respectively, while only 0.008±0.008 g·m^-2^·d^-1^ and 0.010±0.008 g·m^-2^·d^-1^ at sites 4 and 5, being lowest at the sites of highest macrophyte coverage (site 4). The TP resuspension rates at sites 1, 2, 3, and 6 were significantly higher than at sites 4 and 5 (*Z* = −6.087, *p* < 0.01).

### Wind speed and direction

Annual average wind speed at site 1 was 3.1 m·s^-1^, and the dominant wind direction was from the east. Monthly averaged wind speed ranged from 2.5 m·s^-1^ (October 2012) to 3.8 m·s^-1^ (August 2012). The dominant wind direction was from the southeast in summer and from the north in winter. Taihu was influenced by Typhoon Haikui in Aug 2012, and the highest wind speed on 8 Aug 2012 was 19.1 m·s^-1^. At sites 4 and 5, the annual averaged wind speed was 2.7 m·s^-1^, and the dominant wind direction was from the southeast.

**Fig 6:**
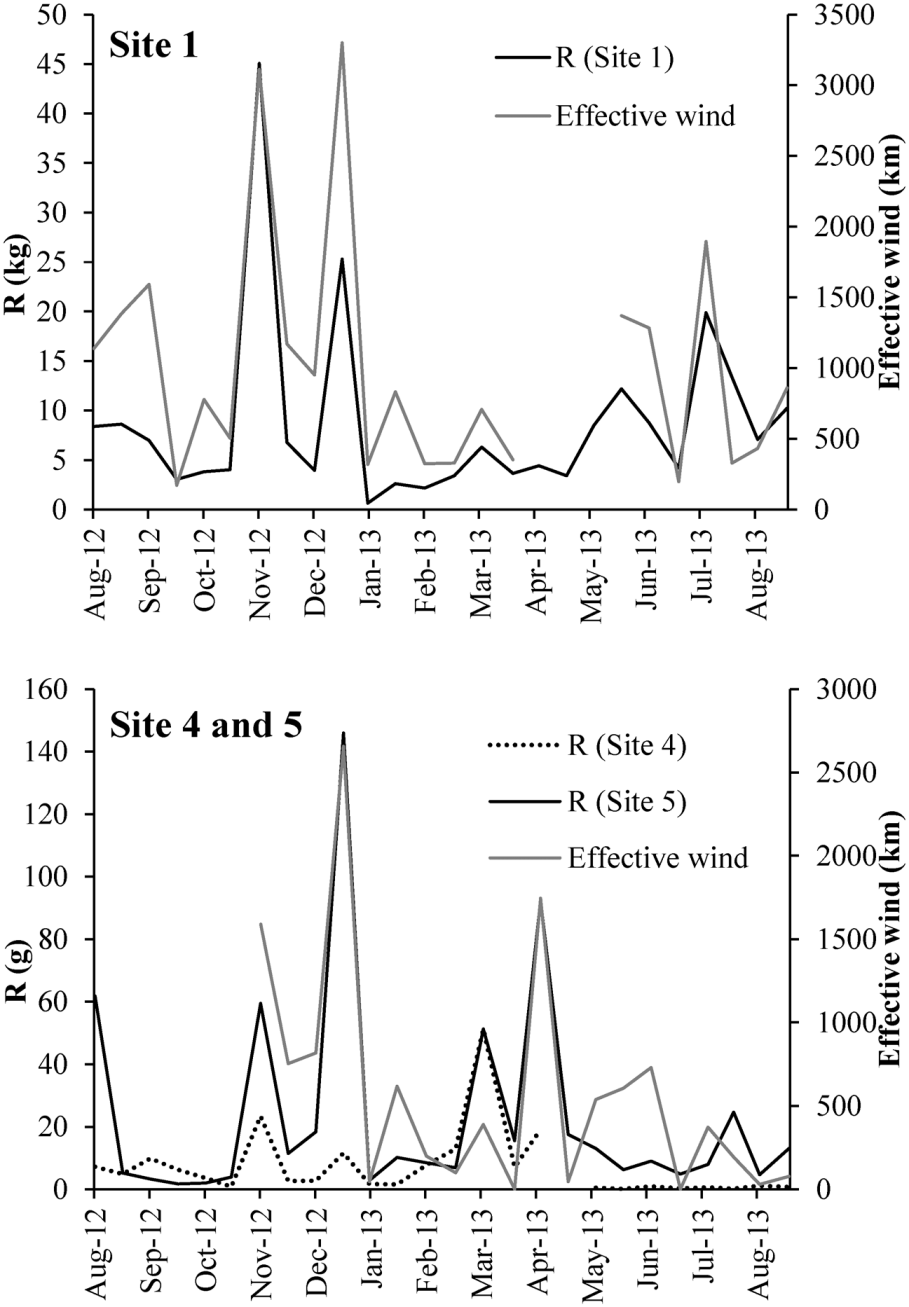
Effective wind and resuspended sediment at sites 1, 4 and 5 during the observation period.

Effective wind had highly significant correlation with resuspended sediment at phytoplankton-dominated site 1 (Fig 6, *R* ^*2*^ = 0.712, *p* < 0.01) and at macrophyte semi-covered site 5 (Fig 6, *R* ^*2*^ = 0.807, *p* < 0.01), but not at macrophyte fully-covered site 4 (Fig 6, *R* ^*2*^ = 0.084, *p* > 0.05).

## Discussion

### The influence of lake morphology on wind-induced resuspension

In both phytoplankton- and macrophyte-dominated zones, sediment resuspension correlated significantly with effective wind, indicating that wind speed is one of the most important factors causing sediment resuspension in large and shallow lakes [51,52], especially long-lasting sustained strong wind [16]. The annual average sediment resuspension rate at the most offshore site (site 3) was nearly 2 times higher than at the lakeshore site (site 1), which was mostly due to the different wind fetch. Site 3 obviously has the longest distance summation to shoreline, and site 1 has the shortest (Table 3). Therefore, located in the central part of the large lake, site 3 experienced more wind from each direction than the sites in the bay, and thus also the sediment resuspension rate was higher.

**Table 3:** The distances from each site to the shorelines in four directions (km).

Sites	East	South	West	North	Summation
**1**	0	1	8	0	9
**2**	11	48	13	5	77
**3**	22	36	22	29	109
**4**	1	1	24	8	34
**5**	1	1	24	9	35
**6**	2	6	8	3	19

**Fig 7:**
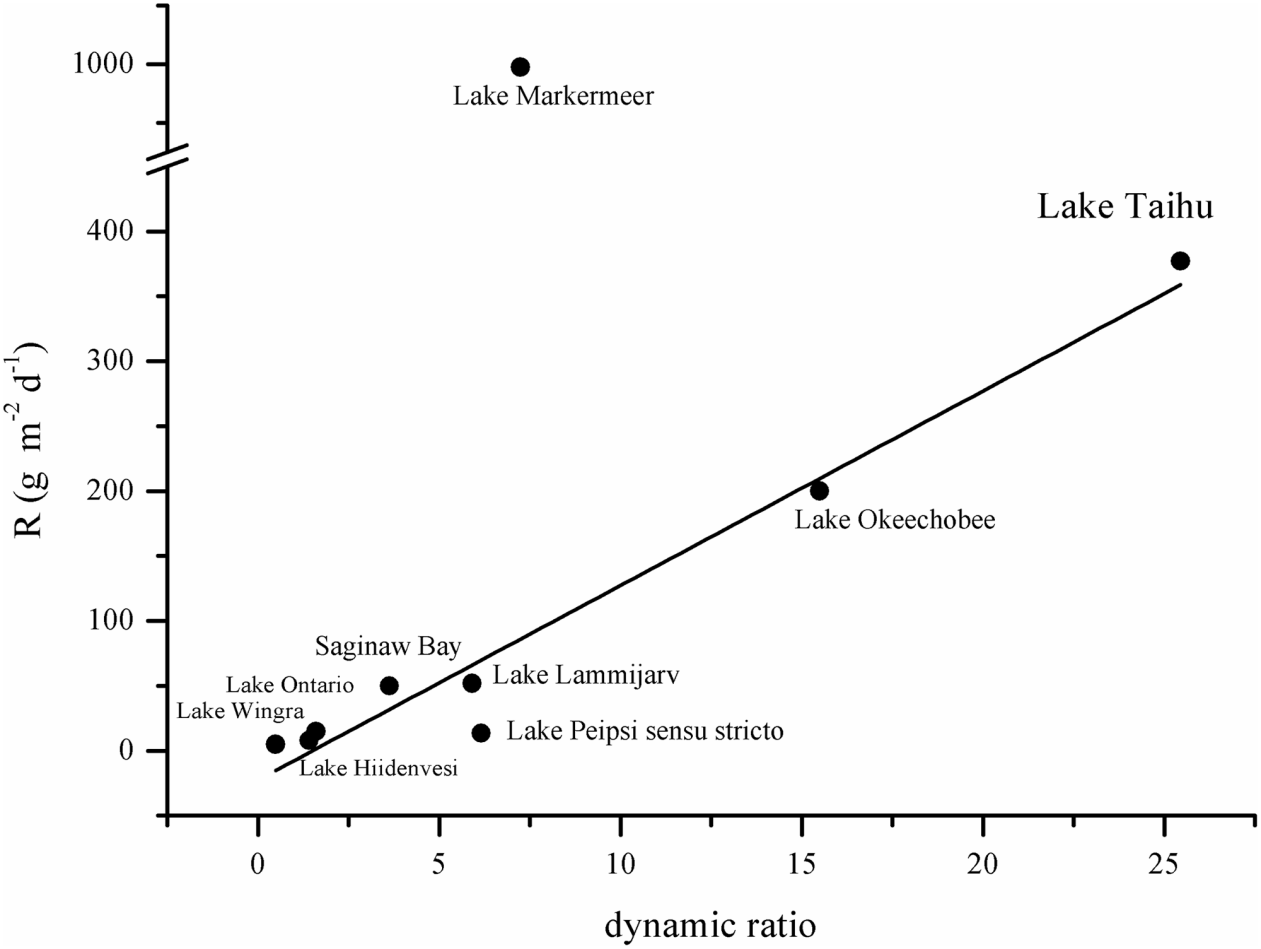
Dynamic ratio and sediment resuspension rate (R) in different lakes. Peak suspended solid concentrations during resuspension were used for Saginaw Bay and Lake Okeechobee instead of sediment resuspension rate (R) data in this figure, as there was no detailed sediment resuspension rate data in the references [52,53]. The trend line showing the relationship between R and dynamic ratio was calculated excluding Lake Markermeer.

However, under similar wind conditions, sediment resuspension varies strongly depending on lake morphology. Sediment resuspension is prone to occur in lakes with low water depth and large open areas for wind fetch [11,12]. Taihu is very shallow and has quite large surface area, resulting in higher dynamic ratio and sediment resuspension rate. Lakes with smaller dynamic ratios have been shown to have lower sediment resuspension rates (Fig 7), such as shallow but small Lake Wingra, Lake Hiidenvesi and Lake Lammijarv [8,11,21], or large but deep Lake Peipsi, Saginaw Bay and Lake Okeechobee [8,52,53], or even a larger lake such as Lake Ontario [11]. Lake Markermeer also has a smaller dynamic ratio than Taihu, but a higher estimated annual sediment resuspension rate (Fig 7). This is mostly due to stronger near-bottom currents (> 10 cm·s^-1^) [22], while currents at site 1 in Taihu ranged from 0.2 to 3.3 cm·s^-1^, averaging 1.5 cm·s^-1^ [16].

### The influence of ecotype on sediment resuspension

Macrophytes showed a stronger influence on the variation of sediment resuspension in different regions than wind fetch in Taihu. The summation of distances from both macrophyte-dominated sites 4 and 5 to the shoreline in four directions is 34 and 35 km, which is nearly 2-fold longer than at site 6 and nearly 4-fold longer than at site 1 (Table 3), but the annual average sediment resuspension rates at sites 4 and 5 were 29-fold lower than corresponding rates at sites void of macrophyte (sites 1, 2, 3, and 6). Therefore, the presence of macrophytes showed a substantial effect on reducing sediment resuspension in Taihu, as proved in other studies in shallow waters [21], for vegetation reduces the wind-induced waves [20,54]. The annual average sediment resuspension rate at macrophyte fully covered site 4 was thus lowest of all sites, showing no correlation to wind speed.

**Fig 8:**
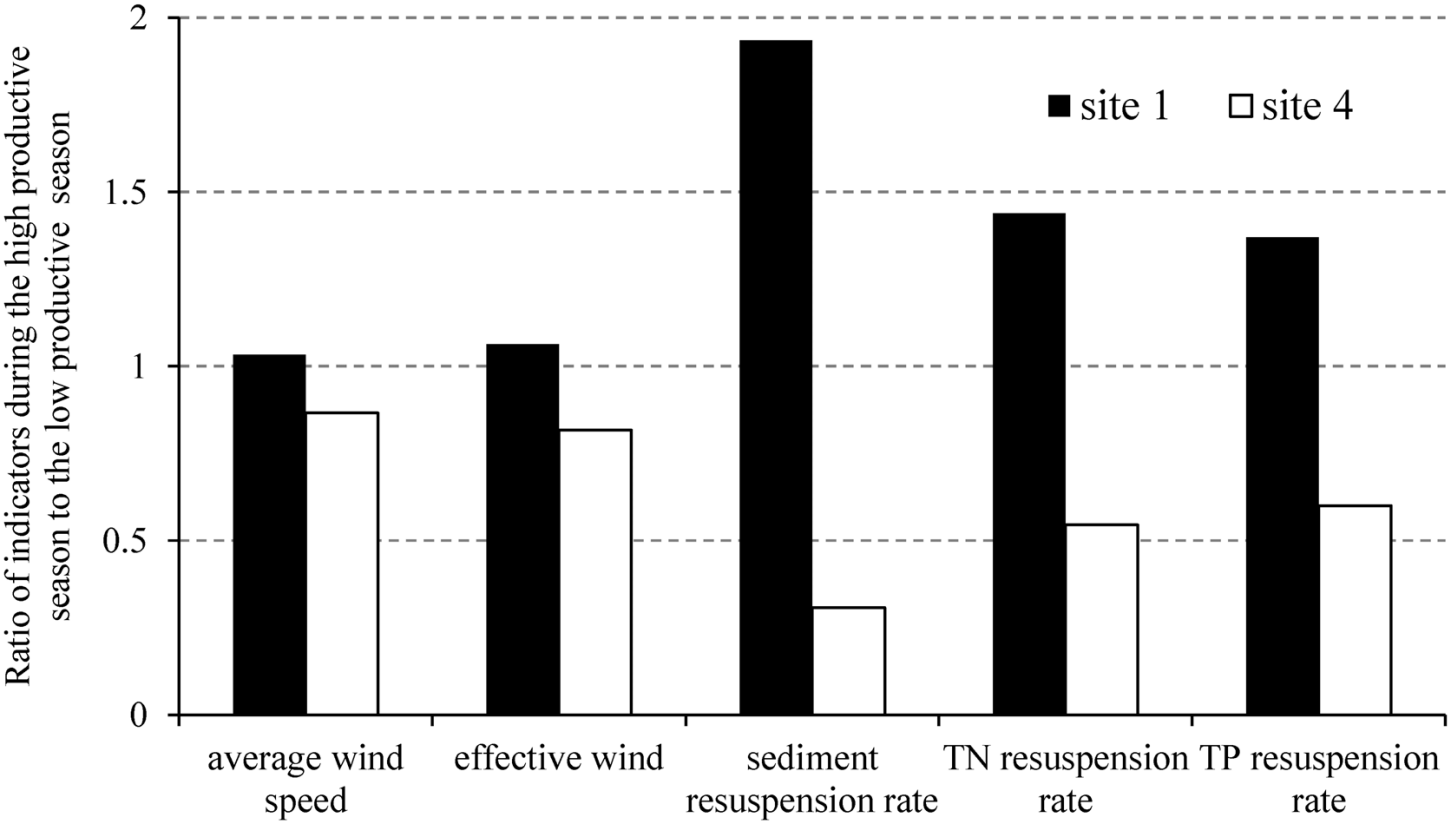
Ratios of average wind speed, effective wind, sediment resuspension rate, total nitrogen (TN) and total phosphorus (TP) resuspension rate during the high productive season to the low productive season at sites 1 and 4.

Sediment resuspension also showed variation between the high productive and the low productive seasons in the macrophyte-dominated zone. The ratio of sediment resuspension rates at macrophyte-dominated sites 4 and 5 during the high productive season to that during the low productive season was only 0.31 and 0.39 (Fig 8). Lower wind speed was an important factor for a lower sediment resuspension rate during the high productive season, but the averaged wind speeds at sites 4 and 5 during the high productive and the low productive seasons were 2.6 m·s^-1^ and 3.0 m·s^-1^, respectively, not showing considerable differences. The ratio of effective wind at sites 4 and 5 during the high productive season to that during the low productive season was 0.82 (Fig 8), indicating that, besides wind speed, the growth and degradation of macrophytes during different seasons was also a very important factor reducing sediment resuspension. During the high productive season, the suspended matter was less, mostly consisted of leaf blades, having larger average median particle size (9.3 μm). However, during the low productive season, containing more inorganic matter, the concentration of suspended solids was higher, with smaller average median particle size (7.5 μm).

In the phytoplankton-dominated zone, the existence of blooms also caused the sediment resuspension rate to vary during the high productive and the low productive seasons. The ratio of sediment resuspension rate at phytoplankton-dominated site 1 during the high productive season to that during the low productive season was 1.94, but the averaged wind speeds during the high productive season and the low productive season were 3.1 m·s^-1^ and 3.0 m·s^-1^, respectively, not showing any difference (Fig 8). The ratio of effective wind at site 1 during the high productive season to that during the low productive season was only 1.06, which additionally supported the effect of phytoplankton blooms on sediment resuspension.

The presence of blooms may influence sediment resuspension for several reasons. The accumulation and sedimentation of phytoplankton blooms increase the amount of particulate matter in the water column and sediment surface, which may easily disaggregate and be resuspended [55]. Phytoplankton blooms may capture small particles when sinking or resuspending, as phytoplankton excrete slime [56,57]. Phytoplankton blooms in the water column and on the sediment surface may also increase the organic matter content, aggregate inorganic matter and be easily resuspended.

The annual average sediment resuspension rate in the phytoplankton-dominated zone estimated in this study, based on a one-year observation, was lower than the results estimated by Huang and Liu [32] from June to September. It is mainly because sediment in that study site had a higher organic matter content (5.93–10.53%), and also because that study was conducted only in the high productive season, which may overestimate the annual average value, due to the significant seasonal variation of sediment resuspension rate.

### Nutrient supply to the water column by sediment resuspension

Sediment resuspension is an important source for internal nutrient loading in shallow lakes [4,19,26], and our data confirm this is also the case in Taihu: Indeed the nutrient resuspension rate in Taihu was higher compared to other lakes. Phosphorus resuspension rates were estimated as 0.060 to 0.070 g·m^-2^·d^-1^ in Lake Arresø [4], and 0.039 g·m^-2^·d^-1^ in Lake Hiidenvesi [21], which were much lower than the TP resuspension rate in Taihu’s phytoplankton-dominated zone (0.142 g·m^-2^·d^-1^), although higher than in the macrophyte covered zone (0.009 g·m^-2^·d^-1^).

TN and TP concentrations in the water column may be increased by 0.34 mg·L^-1^ and 0.051 mg·L^-1^ on average caused by sediment resuspension in areas void of macrophytes, while they may only be increased by 0.03 mg·L^-1^ and 0.003 mg·L^-1^ in macrophyte covered areas, respectively. In addition, the increased nutrient concentration in the water column calculated in the macrophyte-dominated zone might be overestimated, as the large and extensive macrophyte coverage in the southeastern lake should have stronger effect in reducing resuspension rate than the small pieces of macrophyte coverage at sites 4 and 5. Thirty percent of the TP content in surface sediment is in algal-available forms [48]. The average equilibrium concentrations of nitrogen and phosphorus at the sediment-water interface in Taihu were 1.10 mg/L and 0.11 mg/L, respectively, and the concentrations were much higher in areas with frequent bloom occurrence [58]. Therefore, nutrients stored in the sediment in Taihu still have great potential to be released into the water column, as an important source for phytoplankton growth.

TN and TP resuspension rates at site 1 was higher than site 2, mostly due to the higher TOC content in the sediment at site 1. The reason for higher TOC content at the lakeshore site 1 was the more efficient source for organic matter, such as submerged plants or reed. Therefore, sediment properties, especially the organic matter content, had an important influence on nutrient resuspension.

The amount of organic matter in suspended sediment was even more important than total sediment resuspension for nutrient resuspension. The annual average TN and TP resuspension rate in the areas void of macrophytes (sites 1, 2, 3 and 6) was 10-fold and 16-fold the resuspension rate in the macrophyte covered areas (sites 4 and 5), respectively. The differences in the nutrient resuspension rates between non-macrophyte and macrophyte-covered areas were not as clear as differences between sediment resuspension rates (29-fold), but similar to the differences in the resuspension rates of organic matter (14-fold).

Similar to the sediment resuspension rate, nutrient resuspension rate also varied between the high productive and the low productive season. At macrophyte fully-covered site 4, the ratios of TN and TP resuspension rates during the high productive season to the low productive season were 0.55 and 0.60 (Fig 8), higher than the ratio of sediment resuspension rate (0.31) but lower than the ratio of effective wind (0.82). At phytoplankton-dominated site 1, the ratios of TN and TP resuspension rates during the high productive season to the low productive season were 1.44 and 1.37 (Fig 8), lower than the ratio of sediment resuspension rate (1.94) but higher than the ratio of effective wind (1.06).

### Sediment resuspension promotes phytoplankton growth

Sediment resuspension may add on average 161 mg·L^-1^ of suspended solids in the water column in open water areas, while only 6 mg·L^-1^ of suspended solids in macrophyte-covered areas. The resuspended sediment may settle down when wind speed decreases and water flow slows down, but the increment of SS concentration during strong wind periods may significantly influence the transparency in the water column. According to the relationship between secchi depth and SS concentration [59] and the regression relationship between the photosynthetically available radiation (PAR) euphotic depth and SS concentration in Taihu [60], when SS concentration is 161 mg·L^-1^, the corresponding secchi depth is only 14 cm and PAR euphotic depth is 39 cm. In the macrophyte-covered zone, the calculated secchi depth is over 1 meter and PAR euphotic depth is over 2 meters, even deeper than the water depth at sites 4 and 5, which indicated that macrophytes greatly influenced transparency and the underwater light field.

However, once macrophytes disappear, the lower transparency in the water column caused by sediment resuspension would limit the recolonization of macrophytes. In Taihu, summer blooms are dominated by the buoyant cyanobacteria *Microcystis* spp., which prefers to accumulate at the water surface [39], thus the occurrence of blooms are not affected by reduced light penetration. Due to low transparency, most of the offshore parts of Taihu have no history of macrophyte colonization, not even when eutrophication was not as severe. The restoration of macrophytes could only be considered in certain regions like the lakeshore, and sediment resuspension should be reduced during restoration to ensure colonization using wave barriers or enclosures [32].

Sediment resuspension also brings considerable amounts of nutrients into the water column. TP concentration in Taihu during the 1990s has reported to be above 0.080 mg·L^-1^ [24], and during this study TP concentration in the water column was 0.079 mg·L^-1^ in average for all 6 sites and 0.106 mg·L^-1^ in average for 3 sites in the phytoplankton-dominated zone. The amount of phosphorus resuspended from the sediment in the zone void of macrophytes was comparable with the TP concentration in the water column, and indicates a trophic state index of 61 (in a scale of 0–100) according to Carlson [61]. The trophic state index calculated for TP concentration showed a continuous high trophic level in Taihu, and would not decrease as long as resuspended sediment showed such significant influence. This influence of sediment resuspension on phosphorus concentration in the water column should be considered in lake management and restoration target determination.

At present, sediment resuspension in the macrophyte-dominated zone is low and does not affect water quality. However, the TN and TP contents in the sediment at macrophyte fully covered area (site 4) are much higher than other sites, and the distance summation from site 4 to shorelines are also longer than at sites 6 and 1. Once phytoplankton blooms become dominant, site 4 may face very strong sediment resuspension without the inhibiting effect of macrophytes, similar to site 6, where the shift from macrophyte-dominated zone to non-macrophyte zone has occurred.

## Conclusion

Sediment resuspension in Taihu, especially in the phytoplankton-dominated zone, is much stronger than in many other lakes worldwide. Lake morphology is the key factor for the high sediment resuspension rate in Taihu, as it is very shallow and large to ensure long wind fetch. However, sediment resuspension in different areas of the lake varied a lot due to the ecotype. Under similar wind speed, the growth of macrophytes may reduce sediment resuspension rate by 29-fold according to the annual observation.

Sediment resuspension brought numerous nutrients into the water column and correspondingly reduced transparency. The low transparency prevented the growth or recovery of macrophytes, but benefited the blooming of *Microcystis* spp. in Taihu. The strong and fast cycling of nutrients at the sediment-water interface caused by sediment resuspension kept the phosphorus concentrations in the lake water at a high trophic level in Taihu and stimulated phytoplankton growth.

## References

[1] James WF, Best EP, Barko JW (2004) Sediment resuspension and light attenuation in Peoria Lake: can macrophytes improve water quality in this shallow system? Hydrobiologia 515: 193–201.

[2] Zhang YL, Qin BQ, Zhu GW, Gao G, Luo LC, Chen WM (2006) Effect of sediment resuspension on underwater light field in shallow lakes in the middle and lower reaches of the Yangtze River: A case study in Longgan Lake and Taihu Lake. Sci China Ser D 49: 114–125.

[3] Hofmann H, Lorke A and Peeters F (2008) Wave-induced variability of the underwater light climate in the littoral zone. Int Ver Theor Angew 30: 627–632.

[4] Søndergaard M, Kristensen P, Jeppesen E (1992) Phosphorus release from resuspended sediment in the shallow and wind-exposed Lake Arresø, Denmark. Hydrobiologia 228: 91–99.

[5] Niemisto J, Holmroos H, Pekcan-Hekim Z, Horppila J (2008) Interactions between sediment resuspension and sediment quality decrease the TN:TP ratio in a shallow lake. Limnol Oceanogr 53: 2407–2415.

[6] Nurminen L, Horppila J (2009) Life form dependent impacts of macrophyte vegetation on the ratio of resuspended nutrients. Water Res 43: 3217–3226. 10.1016/j.watres.2009.04.041 19505709

[7] Schallenberg M, Burns CW (2004) Effects of sediment resuspension on phytoplankton production: teasing apart the influences of light, nutrients and algal entrainment. Freshwater Biol 49: 143–159.

[8] Tammeorg O, Niemisto J, Mols T, Laugaste R, Panksep K, Kangur K (2013) Wind-induced sediment resuspension as a potential factor sustaining eutrophication in large and shallow Lake Peipsi. Aquat Sci 75: 559–570.

[9] Bachmann RW, Hoyer MV, Canfield DE (1999) The restoration of Lake Apopka in relation to alternative stable states. Hydrobiologia 394: 219–232.

[10] Dzialowski AR, Wang SH, Lim NC, Beury JH, Huggins DG (2008) Effects of sediment resuspension on nutrient concentrations and algal biomass in reservoirs of the Central Plains. Lake Reserv Manage 24: 313–320.

[11] Evans RD (1994) Empirical-evidence of the importance of sediment resuspension in lakes. Hydrobiologia 284: 5–12.

[12] Scheffer M (1998) Ecology of shallow lakes. Chapman and Hall 313 p.

[13] Bengtsson L, Hellstrom T (1992) Wind-induced resuspension in a small shallow lake. Hydrobiologia 241: 163–172.

[14] Zhang Y, Shi K, Liu X, Zhou Y, Qin B (2014) Lake topography and wind waves determining seasonal-spatial dynamics of total suspended matter in turbid Lake Taihu, China: Assessment using long-term high-resolution MERIS data. PLoS ONE 9: e98055 10.1371/journal.pone.0098055 24846206 PMC4028274

[15] Qin BQ, Hu WP, Gao G, Luo LC, Zhang JS (2004) Dynamics of sediment resuspension and the conceptual schema of nutrient release in the large shallow Lake Taihu, China. Chinese Sci Bull 49: 54–64.

[16] Wu TF, Qin BQ, Zhu GW, Zhu MY, Li W, Luan CM (2013) Modeling of turbidity dynamics caused by wind-induced waves and current in the Taihu Lake. Int J Sediment Res 28: 139–148.

[17] Reardon KE, Bombardelli FA, Moreno-Casas PA, Rueda FJ, Schladow SG (2014) Wind-driven nearshore sediment resuspension in a deep lake during winter. Water Resour Res 50: 8826–8844.

[18] McMullen KY, Poppe LJ, Parker CE (2015) Character, distribution, and ecological significance of storm wave-induced scour in Rhode Island Sound, USA. Geo-Mar Lett 35: 135–144.

[19] Horppila J and Nurminen L (2003) Effects of submerged macrophytes on sediment resuspension and internal phosphorus loading in Lake Hiidenvesi (southern Finland). Water Res 37: 4468–4474. 14511717 10.1016/S0043-1354(03)00405-6

[20] Horppila J, Kaitaranta J, Joensuu L, Nurminen L (2013) Influence of emergent macrophyte (*Phragmites australis*) density on water turbulence and erosion of organic-rich sediment. J Hydrodyn 25: 288–293.

[21] Horppila J, Nurminen L (2001) The effect of an emergent macrophyte (*Typha angustifolia*) on sediment resuspension in a shallow north temperate lake. Freshwater Biol 46: 1447–1455.

[22] Kelderman P, Ang'weya RO, De Rozari P, Vijverberg T (2012) Sediment characteristics and wind-induced sediment dynamics in shallow Lake Markermeer, the Netherlands. Aquat Sci 74: 301–313. 10.2166/wst.2012.325 22925873

[23] Bachmann RW, Hoyer MV, Canfield DE (2000) The potential for wave disturbance in shallow Florida lakes. Lake Reserv Manage 16: 281–291.

[24] Qin BQ (2008) Lake Taihu, China Springer 339 p.

[25] James RT, Havens K, Zhu GW, Qin BQ (2009) Comparative analysis of nutrients, chlorophyll and transparency in two large shallow lakes (Lake Taihu, PR China and Lake Okeechobee, USA). Hydrobiologia 627: 211–231.

[26] Qin BQ, Zhu GW, Luo LC, Gao G, Gu BH (2006) Estimation of internal nutrient release in large shallow Lake Taihu, China. Sci China Ser D 49: 38–50.

[27] Guo L (2007) Doing battle with the green monster of Taihu Lake. Science 317: 1166–1166. 17761862 10.1126/science.317.5842.1166

[28] Qin BQ, Zhu GW, Gao G, Zhang YL, Li W, Paerl HW, et al (2010) A drinking water crisis in Lake Taihu, China: Linkage to climatic variability and lake management. Environ Manage 45: 105–112. 10.1007/s00267-009-9393-6 19915899

[29] Paerl HW, Fulton RS, Moisander PH, Dyble J (2001) Harmful freshwater algal blooms, with an emphasis on cyanobacteria. Sci World 1:76–113. 10.1100/tsw.2001.16 PMC6083932 12805693

[30] Liu XH, Zhang YL, Yin Y, Wang MZ, Qin BQ (2013) Wind and submerged aquatic vegetation influence bio-optical properties in large shallow Lake Taihu, China. J Geophys Res-Biogeo 118: 713–727.

[31] Qian J, Zheng SS, Wang PF, Wang C (2011) Experimental study on sediment resuspension in Taihu Lake under different hydrodynamic disturbances. J Hydrodyn 23: 826–833.

[32] Huang PS, Liu ZW (2009) The effect of wave-reduction engineering on sediment resuspension in a large, shallow, eutrophic lake (Lake Taihu). Ecol Eng 35: 1619–1623.

[33] Kaitaranta J, Niemisto J, Buhvestova O, Nurminen L (2013) Quantifying sediment resuspension and internal phosphorus loading in shallow near-shore areas in the Gulf of Finland. Boreal Environ Res 18: 473–487.

[34] Lowe EF, Battoe LE, Coveney MF, Schelske CL, Havens KE, Marzolf ER, et al (2001) The restoration of Lake Apopka in relation to alternative stable states: an alternative view to that of Bachmann et al. (1999). Hydrobiologia 448: 11–18.

[35] Jeppesen E, Jensen JP, Sondergaard M, Hansen KS, Moller PH, Rasmussen HU, et al (2003) Does resuspension prevent a shift to a clear state in shallow lakes during reoligotrophication? Limnol Oceanogr 48: 1913–1919.

[36] Penning WE, Genseberger M, Uittenbogaard RE, Cornelisse JC (2013) Quantifying measures to limit wind-driven resuspension of sediments for improvement of the ecological quality in some shallow Dutch lakes. Hydrobiologia 710: 279–295.

[37] Chen FZ, Shu TT, Jeppesen E, Liu ZW, Chen YW (2013) Restoration of a subtropical eutrophic shallow lake in China: effects on nutrient concentrations and biological communities. Hydrobiologia 718: 59–71.

[38] Dong BL (2011) Responses to habitat heterogeneity of submerged macrophyte communities and their environmental factors in Lake Taihu. The postdoctoral graduation thesis of Nanjing Institute of Geography and Limnology, Chinese Academy of Sciences (in Chinese with English abstract).

[39] Chen YW, Qin BQ, Teubner K, Dokulil MT (2003) Long-term dynamics of phytoplankton assemblages: *Microcystis*-domination in Lake Taihu, a large shallow lake in China. J Plankton Res 25: 445–453.

[40] Ma RH, Duan HT, Gu XH, Zhang SX (2008) Detecting aquatic vegetation changes in Taihu Lake, China using multi-temporal satellite imagery. Sensors-Basel 8: 3988–4005. 27879919 10.3390/s8063988 PMC3924936

[41] Heiri O, Lotter AF, Lemcke G (2001) Loss on ignition as a method for estimating organic and carbonate content in sediments: reproducibility and comparability of results. J Paleolimnol 25: 101–110.

[42] Jin XC, Tu QY (1990) Standard of lake eutrophication survey of China. China Environmental Science Publishing House 317 p (in Chinese).

[43] Qian JL, Zhang LD, Le ML (1990) Determination of total nitrogen and total phosphorus in soils by persulfate digestion. Soils 22: 258–262 (in Chinese).

[44] Zhang W, Xu B (1984) Study methods for forest soil. China Forestry Publishing House 186 p (in Chinese).

[45] Jespersen AM, Christoffersen K (1987) Measurements of chlorophyll-a from phytoplankton using ethanol as extraction solvent. Arch Hydrobiol 109: 445–454.

[46] Gasith A (1975) Tripton sedimentation in eutrophic lakes—Simple correction for the resuspended matter. Verhandlungen der Internationalen Vereinigung für Limnologie 19: 116–122.

[47] Ma RH, Duan HT, Tang JW, Chen ZB (2010) Remote sensing of water environment in lakes. Science Press 536 p (in Chinese).

[48] Zhu MY, Zhu GW, Li W, Zhang YL, Zhao LL, Gu Z (2013) Estimation of the algal-available phosphorus pool in sediments of a large, shallow eutrophic lake (Taihu, China) using profiled SMT fractional analysis. Environ Pollut 173: 216–223. 10.1016/j.envpol.2012.10.016 23202653

[49] Luo LC, Qin BQ, Zhu GW (2004) Calculation of total and resuspendable sediment volume in Lake Taihu. Oceanol Et Limnol Sin 35: 491–496 (in Chinese with English abstract).

[50] Zhang Y, Qin B, Chen W, Luo L (2004) A study on total suspended matter in Lake Taihu. Resour Environ Yangtze Basin 13: 266–271 (in Chinese with English abstract).

[51] Li Y, Pang Y, Li Y (2007) Resuspended flux of sediment in Taihu Lake under hydrodynamic action. Shuili Xuebao 38: 558–564 (in Chinese with English abstract).

[52] Jin KR, Sun DT (2007) Sediment resuspension and hydrodynamics in Lake Okeechobee during the late summer. J Eng Mech-Asce 133: 899–910.

[53] Hawley N, Redder T, Beletsky R, Verhamme E, Beletsky D and DePinto JV (2014) Sediment resuspension in Saginaw Bay. J Great Lakes Res 40: 18–27.

[54] Madsen JD, Chambers PA, James WF, Koch EW, Westlake DF (2001) The interaction between water movement, sediment dynamics and submersed macrophytes. Hydrobiologia 444: 71–84.

[55] Auffret G, Khripounoff A, Vangriesheim A (1994) Rapid post-bloom resuspension in the northeastern Atlantic. Deep-Sea Res Pt I 41: 925–939.

[56] Lapasin R, Pricl S, Bertocchi C, Navarini L, Cesaro A, Dephilippis R (1992) Polysaccharides from Cyanobacteria. 4. Rheology of culture broths and exopolysaccharide of *Cyanospira-Capsulata* at different stages of growth. Carbohyd Polym 17: 1–10.

[57] Huang Z, Liu Y (1997) Polysaccharides from cyanobacteria and their potential applications. Biotechnol Bull 4: 26–32 (in Chinese with English abstract).

[58] Jiang X, Wang QJ, Wang SH, Jin XC, Li YF (2011) Characteristic analysis of the adsorption/desorption of nitrogen and phosphorus in the sediments of Taihu Lake. Environ Sci 32: 1285–1291 (in Chinese with English abstract). 21780581

[59] Zhang Y, Qin B, Chen W, Hu W, Yang D (2003) Distribution, seasonal variation and correlation analysis of the transparency in Taihu Lake. J Oceanol Limnol 2: 30–36 (in Chinese with English abstract).

[60] Zhang YL, Qin BQ, Hu WP, Wang SM, Chen YW, Chen WM (2006) Temporal-spatial variations of euphotic depth of typical lake regions in Lake Taihu and its ecological environmental significance. Sci China Ser D 49: 431–442.

[61] Carlson RE (1977) Trophic state index for lakes. Limnol Oceanogr 22: 361–369.

